# Assessing the policy and institutional framework for multisectoral governance and accountability in emergency preparedness and response in Ethiopia

**DOI:** 10.1371/journal.pgph.0006915

**Published:** 2026-07-24

**Authors:** Sileshi Demelash Sasie, Melkamu Asrat Alava, Lensa Fekadu, Haymanot Firdie, Neima Zeynu Ali, Tarekegn Solomon, Melkamu Abte Afele

**Affiliations:** Ethiopian Public Health Institute, Addis Ababa, Ethiopia; The University of British Columbia, CANADA

## Abstract

Public health emergencies place sustained pressure on health systems, particularly where effective coordination across sectors and levels of governance is required. Although Ethiopia has established policy frameworks and coordination mechanisms aligned with global initiatives, evidence on how these arrangements function in practice remains limited. This study assessed multisector governance for public health emergency preparedness and response, focusing on accountability, coordination, institutional roles, and implementation. A sequential mixed-methods study was conducted in Ethiopia between July and September 2025. Quantitative data were collected through a cross-sectional survey of 74 stakeholders from institutions involved in emergency preparedness and response across the health, disaster risk management, agriculture, livestock, academic, and partner sectors. Governance was assessed across seven predefined domains using 5-point Likert-scale measures, with domain scores summarized using descriptive institutional maturity categories. Qualitative data from purposively selected key informants were analyzed thematically to contextualize and explain quantitative findings. The survey achieved a response rate of 92.5%. The overall perceived governance maturity score was 3.31, corresponding to the predefined functional category. Most governance domains were rated as functional, whereas policy framework implementation remained at the emerging level. Qualitative findings indicated that national guidelines and coordination platforms were widely recognized, but their implementation varied across administrative levels. Persistent challenges included role ambiguity, inconsistent coordination and reporting practices, weak feedback mechanisms, and uneven implementation of policy and legal frameworks, particularly at subnational levels, where governance frequently relied on adaptive and informal practices. Overall, Ethiopia has established a functional institutional foundation for multisector emergency governance; however, important policy-to-practice gaps remain. Strengthening operational guidance, coordination, accountability, and implementation across administrative levels will be essential to improve emergency preparedness and response.

## 1. Background

Public health emergencies, including pandemics, natural disasters, and armed conflicts, place sustained strain on health systems and expose the strengths and weaknesses of multisector governance. They test whether institutions can coordinate across sectors, allocate resources under pressure, and maintain accountability across administrative levels [1–4]. In many settings, emergencies disrupt essential health services, overwhelm routine system capacity, and reveal long-standing gaps in preparedness and surveillance. The COVID-19 pandemic made these dynamics unmistakable. Maternal and child health services, HIV and tuberculosis care, and chronic disease management were widely interrupted, with 90–94% of countries reporting disruptions to essential health services [5–8]. These interruptions were associated with increased morbidity and mortality and threatened to reverse decades of progress in reducing preventable deaths and controlling infectious diseases [7,8]. Their effects were not evenly distributed. Existing social and economic inequities intensified the impact of emergencies, with women, children, people with disabilities, and populations living in poverty experiencing disproportionate direct and indirect consequences [5,6,9].

In response to the growing frequency and complexity of public health emergencies, global, regional, and national actors have developed a range of governance frameworks to strengthen preparedness and response. At the global level, the International Health Regulations (IHR) constitute the primary legal instrument, committing 196 countries to develop and sustain core capacities for surveillance, reporting, and response to public health threats of international concern. The IHR Monitoring and Evaluation Framework (MEF), including State Party Annual Reporting (SPAR), Joint External Evaluation (JEE), After Action Reviews (AAR), and Simulation Exercises (SimEx), is intended to support countries in assessing preparedness, identifying gaps, and prioritizing improvements [10–14]. Beyond technical assessment, the IHR provide a basis for international collaboration, transparency, and collective action, including through the declaration of Public Health Emergencies of International Concern (PHEICs) for events such as COVID-19, Ebola, and Zika [10,11,13,14]. Ongoing reforms to the IHR and negotiations toward a global pandemic accord reflect efforts to address weaknesses exposed during COVID-19, including delayed reporting, inequitable access to countermeasures, and limited compliance and accountability [10,13,15].

Regional coordination mechanisms further shape how countries prepare for and respond to health emergencies. Regional organizations, such as the European Centre for Disease Prevention and Control (ECDC), support member states through technical assistance, cross-border information sharing, and workforce development [16,17]. Complementary approaches, including the One Health framework, seek to bridge human, animal, and environmental health systems, while Public Health Emergency Operations Centers (PHEOCs) are intended to provide a focal point for multisector coordination at national level during preparedness and response, particularly for complex and zoonotic threats [6,10,16,18].

Despite the proliferation of frameworks and coordination platforms, persistent limitations remain across levels of governance. Globally, legally binding instruments offer an essential foundation for collective action, but their implementation is often constrained by weak enforcement, limited accountability, and substantial disparities in financial and technical capacity. Many countries continue to struggle to meet core preparedness and response requirements [2,12,19]. More broadly, the global health security landscape is shaped by fragmented governance arrangements, entrenched sectoral silos, and power imbalances between countries, all of which complicate priority setting and undermine effective cooperation [2,18,20].

Similar challenges are evident at regional and national levels. While regional coordination mechanisms have supported cross-border collaboration, implementation is frequently uneven, with limited integration across sectors and inconsistent alignment with national and subnational priorities [16,21,22]. National preparedness frameworks face their own constraints, including unstable financing, limited workforce capacity, and fragmented coordination across government sectors and with external partners. Together, these factors contribute to uneven levels of readiness and response capability within and across countries [1,23,24]. Across settings, strengthening governance arrangements, securing sustainable investment, improving multisector collaboration, and addressing equity and community engagement remain central to building resilient health systems capable of managing public health emergencies [1,2,16,21,22,25,26].

Against this backdrop, this study draws on recent methodological efforts to systematize the assessment of multisector emergency governance, including the Multi-Sector Emergency Governance Assessment Toolkit and the Guided Assessment for Multi-Sector Governance in Public Health Emergencies. These initiatives emphasize structured, domain-based evaluation of governance arrangements while allowing for contextual adaptation. Using an integrated quantitative and qualitative approach, this paper aims to assess the maturity and performance of multisector governance for public health emergency preparedness and response in Ethiopia, with particular attention to policy and institutional frameworks, coordination mechanisms, accountability arrangements, and implementation processes across sectors and levels of governance. Critically, this study addresses a specific evidence gap: while Ethiopia has established policy frameworks (PHEM guidelines, IMS, One Health platform) and coordination structures, very little empirical research has examined how these governance arrangements function operationally particularly the gap between formal policy design and real-world implementation practices across administrative levels, and how this gap varies during and between emergencies.

## 2. Methods

### 2.1. Study setting and design

This study was conducted in Ethiopia between 10 July and 10 September 2025 across institutions involved in public health emergency preparedness and response at federal, regional, and woreda levels. A sequential mixed-methods design was employed to examine multisector emergency governance, integrating a cross-sectional quantitative survey with qualitative key informant interviews. The quantitative component provided a structured assessment of governance domains across institutions, while the qualitative component was used to explore operational experiences and contextualize quantitative findings.

### 2.2. Study population and sampling

The study population comprised institutions and professionals involved in public health emergency preparedness, response, and governance. These included the Ethiopian Public Health Institute, the Federal Ministry of Health, the Ethiopian Disaster Risk Management Commission, relevant sectoral ministries (agriculture, livestock, and environment), regional and zonal offices, non-governmental organizations, United Nations agencies, and academic institutions. Eligible participants were individuals directly engaged in governance-related functions, including decision-makers, coordinators, technical leads, and subject-matter experts.

Purposive sampling was employed. Institutions involved in public health emergency preparedness and response were first identified through national stakeholder mapping [25], and individuals were then selected within these institutions to ensure representation across sectors, administrative levels, and governance roles.

For the quantitative component, a target sample size of 80 respondents was calculated using a single-population proportion approach, assuming a conservative proportion of 0.5, a 90% confidence level, and a 10% margin of error, with adjustment for anticipated non-response [27,28]. This approach was considered appropriate given the finite and specialized population of multisector emergency governance actors and the descriptive objectives of the analysis. For the qualitative component, 10–20 key informants were purposively recruited from the same institutional pool, with interviews conducted until thematic saturation was achieved [29].

### 2.3. Data collection tools and procedures

Data collection tools were developed specifically for this study, informed by key principles and governance domains outlined in the Multisector Emergency Governance Assessment Toolkit and the Guided Assessment for Multisector Governance in Public Health Emergencies. These sources were used to inform domain selection and item formulation, while the final assessment instrument was tailored to the Ethiopian context and the specific objectives of the study. The complete survey questionnaire and interview guide are provided in the supplementary materials (S1 File).

The seven governance domains were defined a priori based on key principles from the Multisector Emergency Governance Assessment Toolkit and the Guided Assessment for Multisector Governance in Public Health Emergencies. Each domain was operationalized using 4–6 Likert-scale items. For example: the ‘Governance frameworks’ domain included items on clarity of national PHEM guidelines, accessibility of policy documents, and alignment with IHR; the ‘Institutional roles’ domain included items on role clarity across sectors, separation of mandates, and clarity of accountability assignments during emergencies; the ‘Coordination mechanisms’ domain included items on frequency of multisector meetings, clarity of reporting pathways, and use of coordination platforms. A complete domain-item mapping matrix is provided in S1 File.

The quantitative survey instrument (S1 File) comprised two sections. Section A collected information on respondent demographics, institutional characteristics, familiarity with governance frameworks, participation in preparedness and response activities, and routine organizational practices, using structured categorical and binary response items. Section B consisted of a stakeholder perception questionnaire using 5-point Likert-scale items (1 = strongly disagree to 5 = strongly agree), organized across seven governance domains: governance frameworks; institutional roles; coordination mechanisms; preparedness oversight, equity, and transparency; challenges with policy frameworks; strengthening multisector coordination; and implementation strategy.

The qualitative component employed a semi-structured key informant interview guide aligned with the same seven governance domains. Interviews were conducted face-to-face using open-ended questions and probes to elicit participants’ descriptions of institutional practices, coordination processes, implementation experiences, and perceived challenges across sectors and administrative levels.

Prior to data collection, both instruments were reviewed by senior public health emergency management professionals at the Ethiopian Public Health Institute, and minor revisions were made to enhance clarity and usability across sectors and levels of administration. Quantitative data were collected using a self-administered online questionnaire. Qualitative interviews were audio-recorded with participant consent, transcribed verbatim, and supplemented with field notes.

### 2.4. Data analysis

Quantitative data were entered into SPSS (version 28) and analyzed descriptively using frequencies, means, medians, and standard deviations. Domain-level maturity scores were calculated as mean item scores within each governance domain, and an overall institutional maturity score was derived by averaging domain scores. Scores ranged from 1 to 5 and were categorized into four descriptive maturity levels: nascent (1.0–1.9), emerging (2.0–2.9), functional (3.0–3.5), and optimized (3.6–5.0). These categories were applied as a descriptive analytical device to facilitate comparison across governance domains, not as an externally validated performance standard. The cutoff thresholds are consistent with maturity models used in health systems evaluation [30,31].

These categories were defined a priori to facilitate comparative interpretation across governance domains. The maturity classifications were applied as a descriptive analytical device, rather than as a normative benchmark or performance standard. The categorization was adapted from an unpublished national-level governance maturity framework developed for health system and emergency preparedness assessments and is conceptually consistent with maturity-based approaches commonly used in governance and health systems evaluation.

Qualitative data were analyzed thematically using Atlas.ti (version 23). Transcripts were coded iteratively, with codes grouped into higher-order themes aligned with the seven governance domains.

Integration of quantitative and qualitative findings was conducted using a joint display matrix. Following established mixed-methods procedures [32], domain-level quantitative mean scores were compared with qualitatively derived themes. This comparison was structured to assess three types of relationship: convergence (qualitative findings confirming quantitative patterns), divergence (qualitative findings contradicting quantitative patterns), and expansion (qualitative findings providing explanatory depth beyond numerical scores).

Integration of quantitative and qualitative findings occurred during interpretation, with qualitative results used to contextualize and explain observed quantitative patterns, consistent with a sequential mixed-methods approach.

### 2.5. Ethics statement

Ethical approval for this study was obtained from the Hawassa University Institutional Review Board (IRB/536/17; June 30, 2025). Verbal informed consent was obtained from all participants prior to enrollment. For in-person interviews, consent was documented by the data collector using a pre-printed consent log, which recorded the participant’s name, date, and confirmation that consent was freely given. An independent witness (a second data collector not involved in administering that interview) was present during the consent process to verify that information was fully disclosed and that consent was voluntary.

For online survey participants, consent was documented via a required electronic check-box acknowledgement before survey access. This electronic procedure was specifically approved by the IRB as equivalent to written consent. Participation was voluntary, no personally identifiable information was collected, and all data were anonymized and stored securely in password-protected files accessible only to the research team.

## 3. Results

### 3.1. Participant characteristics and governance practices

Of the 80 individuals invited to participate, 74 completed the survey, yielding a response rate of 92.5%. The demographic profile of respondents shows that 59 out of 74(79.7%) were male. Respondents were drawn from a range of institutional settings. Federal ministries and agencies represented the largest share of participants 35(47.3%), followed by regional bureaus 18(24.3%) and zonal health departments 16(21.6%). A smaller proportion of respondents 5(6.8%) were from other institutions.

Respondents demonstrated a broad range of experience in public health emergency management. Thirty-one (42.0%) reported fewer than five years of experience, 30(41.5%) reported five to ten years, and 13(17.5%) reported more than ten years. Regarding functional roles, 37(50.0%) identified as experts, followed by coordinators 18(24.3%), technical leads 13(17.5%), and planners 5(6.8%). Participants were distributed across administrative levels, with 35(47.3%) operating at the national level, 26(35.1%) at the regional level, and 13(17.6%) at the woreda level (Table 1).

**Table 1:** Demographic Characteristics of Respondents (N = 74).

Variable	Category	n(Frequency)	Percent (%)
Sex	Male	59	79.7
	Female	15	20.3
Educational Level	Bachelor’s Degree (BSc/BA)	13	17.6
	Master’s Degree (MSc/MPH/MA)	55	74.3
	MD/DVM	3	4.1
	PhD	3	4.1
Type of Institution Represented	Regional Bureau	18	24.3
	Federal ministry/agency	35	47.3
	Zonal Health Department	16	21.6
	Others	5	6.8
Years of Experience in PHEM	Less than 5 years of work experiences	31	42
	5-10 years of work experiences	30	41.5
	Above 10 work experiences	13	17.5
Primary Role in Emergency Governance	Expert	37	50.0
	Coordinator	18	24.3
	Technical Lead	14	19
	Planner	5	6.8

### 3.2. Governance familiarity, organizational practices, and governance perceptions

Most respondents reported full or partial familiarity with governance frameworks. Awareness of the International Health Regulations and the Sendai Framework was reported by 65(87.8%), and familiarity with the National Incident Management System by 70(94.6%). Attendance at a combined-hazard management workshop within the past two years was reported by 43 (58.1%), and deployment to an emergency response in the previous 12 months by 50(67.6%). Access to emergency policy documents was reported by 54(73.0%), use of digital platforms for PHEM coordination by 52(70.3%), and routine application of standard operating procedures or legal mandates by 53(71.6%). A structured community-engagement mechanism was reported by 57(77.0%).

Regarding governance perceptions, political support for PHEM initiatives was most frequently rated as moderate by 41(55.4%). Resource allocation among sectors was most commonly perceived as partially equitable by 47(63.5%), and trust in inter-agency collaboration was most often rated as moderate by 43(58.1%). External accountability reviews were reported by 33(44.6%) as shown in Table 2.

**Table 2:** Summary of familiarity with governance frameworks, organizational practices, and governance perceptions among respondents (n = 74).

Variable		Response category	Frequency (n)	Percentage (%)
Governance framework familiarity	Familiarity with governance frameworks (IHR, Sendai)	Fully aware	28	37.8
		Somewhat aware	37	50.0
		Not aware	9	12.2
	Familiarity with National Incident Management System (IMS)	Fully aware	41	55.0
		Somewhat aware	29	39.2
		Not aware	4	5.4
Organizational practices related to emergency preparedness and response	Attended combined-hazard management workshop (past 2 years)	Yes	43	58.1
		No	31	41.9
	Deployed to emergency response (past 12 months)	Yes	50	67.6
		No	24	32.4
	Access to emergency policy documents	Yes	54	73.0
		No	20	27.0
	Routine Use of digital platforms for PHEM coordination	Yes	52	70.3
		No	22	29.7
	Routine application of SOPs or legal mandates in PHEM activities	Yes	53	71.6
		No	21	28.4
	External accountability reviews conducted	Yes	33	44.6
		No	41	55.4
	Structured community-engagement mechanism	Yes	57	77.0
		No	17	23.0
Governance perceptions	Political support for PHEM initiatives	High	19	25.7
		Moderate	41	55.4
		Low	14	18.9
	Equity of resource allocation among sectors	Fully equitable	2	2.7
		Partially equitable	47	63.5
		Not equitable	25	33.8
	Trust in inter-agency collaboration	High	14	18.9
		Moderate	43	58.1
		Low	17	23.0

In brief, three main patterns emerged from the integrated analysis. First, the quantitative perceived governance scores across the seven domains ranged from 3.0 to 3.5, with the overall mean perceived maturity score of 3.31 falling within the study’s predefined ‘functional’ descriptive category (3.0–3.5). No domain reached the ‘optimized’ threshold (≥3.6), and the domain addressing ‘challenges with policy frameworks’ scored at the ‘emerging’ level (3.0). Second, the qualitative findings revealed substantial variation in how formal frameworks were applied across administrative levels. Subnational actors consistently reported greater reliance on informal practices, role overlap, and resource constraints compared with federal respondents. Third, policy implementation emerged as the weakest area across both quantitative and qualitative data, with participants citing limited training exposure, unclear operational guidance, and gaps between policy requirements and available capacity as persistent barriers.

### 3.3. Quantitative domain-level perceived governance assessment

Across the seven governance domains assessed, mean scores ranged from 3.0 to 3.5. Governance frameworks, institutional roles, coordination mechanisms, gaps and inconsistencies, strengthening multisector coordination, and implementation strategy were classified at the functional maturity level. The domain addressing challenges with policy frameworks recorded a mean score of 3.0 and was classified as emerging. The mean overall institutional governance maturity score was 3.31, corresponding to a functional level of performance according to the predefined maturity classification. Domain-level scores were calculated by aggregating item-level responses within each domain. The resulting mean scores and corresponding maturity classifications for all domains are presented in Table 3.

**Table 3:** Mean Perceived Scores and Descriptive Maturity Level Across Governance Domains.

Domain	Mean score (SD)	Interpretation
Governance frameworks	3.5 (0.8)	Functional
Institutional role	3.3 (0.9)	Functional
Coordination mechanisms	3.4 (0.9)	Functional
Preparedness Oversight, Equity, and Transparency	3.1 (0.8)	Functional
Challenges with policy frameworks	3.0 (0.8)	Emerging
Strengthening multisector coordination	3.5 (0.8)	Functional

### 3.4. Qualitative findings by governance domain

The qualitative findings draw on data from 10 key informant interviews and are organized into seven thematic categories corresponding to the predefined governance areas. They describe participants’ reported experiences, practices, and observations related to multisector emergency governance across institutions and administrative contexts.

#### 3.4.1. Governance frameworks.

Formally, public health emergency preparedness and response is guided by nationally endorsed governance instruments, including Public Health Emergency Management guidelines, the Incident Management System, early warning and reporting mechanisms, and multisector coordination platforms. Participants described the use of these national governance instruments during emergency preparedness and response. Several participants referred to national PHEM guidelines as the primary reference for routine and emergency activities. One federal participant stated, *“Most of the work we do is according to the national PHEM guideline; it gives us the steps we must follow”* (P1). Another participant described activating the Incident Management System at the onset of emergencies, stating, *“When an emergency starts, the Incident Management System is the first thing we activate because it tells us the structure to use”* (P3).

Operational tools such as early warning bulletins, preparedness plans, and sectoral directives were also described. A disaster risk management officer referred to the early warning bulletin as *“The biggest governance tool we have,”* noting that it requires reporting across sectors (P4). Several participants mentioned the One Health platform as a mechanism used during zoonotic events (P2).

Participants reported variation in how governance frameworks were applied across institutions and administrative levels. A regional participant stated, *“The framework is there, but how we apply it depends on the situation and the leadership at that time”* (P6). Another participant described instances in which actions during acute outbreaks proceeded without reference to written procedures, noting that *“Sometimes the urgency of the situation guides what is done”* (P5).

#### 3.4.2. Institutional roles.

Formally, institutional roles and responsibilities during public health emergencies are defined through national policies, sectoral mandates, and coordination frameworks assigning functions across health, disaster risk management, agriculture, and other sectors. Participants reported that institutional roles are defined in policy documents; however, several described overlapping responsibilities during emergency response. One federal participant stated, *“Roles are written in documents, but during emergencies people cross responsibilities. Everyone wants to help, and this creates overlap”* (P7). Another participant noted that *“Sectors can interpret their roles differently during response”* (P1).

Participants from agriculture and livestock sectors described parallel reporting arrangements between institutions. One participant stated that reporting lines between Agriculture and Health *“create duplication and confusion”* during zoonotic events (P8). A participant involved in risk communication reported uncertainty regarding leadership for community mobilization activities, stating that *“It is not always clear which institution should take the lead”* (P9).

Participants described differences in role clarity depending on coordination arrangements. In some settings, coordination platforms were reported to support clearer task distribution. In other contexts, participants described individuals covering multiple responsibilities. One participant stated, *“In rural areas, one officer may represent the whole department. So roles become more practical than formal”* (P10).

#### 3.4.3. Coordination mechanisms.

Formally, multisector coordination during public health emergencies is intended to operate through established coordination bodies, defined reporting pathways, and scheduled intersectoral meetings linking local, regional, and federal levels. Participants described a range of coordination practices used during emergency preparedness and response. Informal communication methods, including phone calls and messaging applications, were frequently reported. One participant stated, *“Most coordination happens on WhatsApp groups and through phone calls”* (P2). Other participants reported that coordination meetings were mainly convened during major emergencies (P6).

Participants described reporting pathways linking local detection to higher administrative levels, particularly during outbreaks. Federal and regional participants reported receiving information through established reporting channels (P4, P7). At the zonal level, participants described increased reporting frequency during active response periods, including daily briefings (P10).

Participants also reported coordination challenges, including delays in information sharing, inconsistent participation in coordination meetings, and limited follow-up. One participant stated, *“When one sector delays reporting, the whole response slows down”* (P10). Others reported that *“Irregular attendance affects continuity”* (P3, P9).

#### 3.4.4. Preparedness oversight, equity, and transparency.

Formally, emergency governance arrangements include standardized reporting formats, routine evaluations, and feedback mechanisms intended to support accountability and consistency across administrative levels. Participants described variation in reporting practices across administrative levels, including differences in reporting frequency, format, and data completeness. An early warning officer stated that inconsistent reporting across regions leaves *“The national picture incomplete”* (P4). Other participants described differences in the level of detail provided in reports submitted by local administrative units (P1).

Participants also described differences in how standard operating procedures were applied across regions. One participant reported that *“The same SOP is applied differently in different regions”* (P6). In some contexts, participants described reliance on personal communication rather than formal guidance, particularly during zoonotic events (P7). Limited training on guideline use was also reported (P8).

Participants reported limited feedback following implementation of directives. Regional and local participants described carrying out instructions without receiving confirmation or follow-up guidance. One participant stated, *“We implement the directive, but we rarely get feedback on how well it was done”* (P5). Others reported that recommendations from supervisory visits were not always addressed due to resource constraints (P10).

#### 3.4.5. Challenges with policy frameworks.

Formally, policy frameworks are intended to guide implementation through defined mandates, operational procedures, and sustained resource allocation. Participants described difficulties in applying policy frameworks during emergency response. Several participants reported limited exposure to policy-related training. One participant stated that *“Not everyone receives the same level of training, so implementation varies”* (P1). Another participant noted that *“Some policies do not clearly explain how they should be implemented”* (P3).

Resource constraints were frequently reported. Participants from multiple sectors described gaps between policy requirements and available staffing, logistics, and budgets. One participant stated, *“Policies assume you have enough staff and logistics, but on the ground, it is different”* (P9). Similar concerns were reported by participants from livestock and health sectors (P7, P10).

Participants also described overlapping mandates within policy documents. Some reported that coordination is emphasized without detailed clarification of responsibilities where mandates intersect. One participant stated, *“Coordination is mentioned, but roles are not always clear when mandates overlap”* (P5).

#### 3.4.6. Strengthening multisector coordination.

Formally, multisector coordination is expected to be strengthened through joint planning, shared platforms, and structured engagement across sectors and partners. Participants described coordination practices aimed at strengthening multisector engagement during emergency response. Joint response teams were reported as being formed during outbreaks. One participant stated, *“For every outbreak, we create a joint team immediately”* (P2). Other participants described regular coordination calls during response periods (P6).

Formal coordination platforms, including the One Health mechanism, were reported as being used for multisector dialogue, particularly for zoonotic and environmental health events (P4). At the subnational level, participants described joint planning meetings during cholera responses involving the Health, Water, and Education sectors (P10).

Participants also described informal coordination practices, including early engagement with partners and rapid sharing of field information. Academic institutions reported providing technical input during emergencies, stating that *“Evidence is shared quickly to support decision-making”* (P9).

#### 3.4.7. Implementation strategies.

Formally, emergency implementation is expected to follow defined sequences of preparedness, response, and monitoring activities guided by national plans and operational tools. Participants described implementation as involving a sequence of actions that typically included initial risk assessment or mapping, task assignment, and logistical preparation. One participant stated, *“We start with risk assessment, then assign tasks and prepare logistics”* (P1).

Regional and local participants reported reliance on existing staff, facilities, and community structures to carry out response activities (P6). Participants also described the use of monitoring checklists, simulation exercises, and early warning analyses during preparedness and response phases (P2–P4). At local level, implementation practices were described as flexible, including borrowing staff or vehicles from other departments (P7), prioritizing rapid community alerts (P10), and engaging local leaders during response activities (P5).

### 3.5. Domain-level synthesis of qualitative findings across governance dimensions

Key qualitative findings across the seven governance domains assessed are summarized, reflecting recurrent themes reported by participants from multiple institutions and administrative levels. The synthesis describes formal governance arrangements and operational practices as reported in interviews. Detailed participant-level findings and illustrative quotations supporting this synthesis are provided in the online supplementary materials (S1 Table), with a domain-level summary presented in Table 4.

**Table 4:** Summary of key qualitative findings by governance domain.

Governance domain	Summary of key qualitative findings
Governance frameworks	National PHEM guidelines, the Incident Management System, and early warning tools were commonly referenced during emergency preparedness and response. Application of these frameworks varied across institutions and administrative levels, particularly outside formally declared emergencies.
Institutional roles	Roles and responsibilities were described as formally defined in policy documents but frequently overlapped during emergency response. Variations in role interpretation were reported across sectors and administrative levels, especially during zoonotic events.
Coordination mechanisms	Coordination was reported to intensify during active emergencies, supported by established reporting pathways and coordination bodies. Outside emergency periods, coordination relied heavily on informal communication channels, including phone calls and messaging applications.
Gaps and inconsistencies	Participants described variation in reporting practices, application of standard operating procedures, and feedback mechanisms across administrative levels. Differences in reporting completeness and follow-up were commonly reported.
Challenges with policy frameworks	Difficulties in policy implementation were attributed to limited training exposure, resource constraints, and overlapping mandates. Participants reported gaps between policy requirements and available operational capacity.
Strengthening multisector coordination	Joint response teams, One Health platforms, and cross-sector planning meetings were described as mechanisms used to enhance multisector engagement, particularly during zoonotic and environmental health events. Informal coordination practices were also frequently reported.
Implementation strategies	Emergency implementation was described as following pragmatic sequences that included risk assessment, task assignment, and logistical preparation. Adaptation of implementation practices at regional and local levels was reported, including flexible use of staff, resources, and community structures.

### 3.6. Integrated mixed-methods matrix across governance domains

Table 5 integrates the quantitative and qualitative findings. Quantitative scores are means on a 1–5 scale, with descriptive categories in parentheses. Qualitative dominant themes represent the most frequently reported patterns. Dissenting views capture variation by administrative level or sector. Convergence, divergence, and expansion are defined in the Methods.

**Table 5:** Integrated Mixed-Methods Matrix: Quantitative Scores, Qualitative Themes, and Synthesized Interpretation Across Governance Domains.

Domain	Quantitative Score (Mean, descriptive level)	Qualitative Dominant Theme	Qualitative Dissenting or Nuanced Views	Convergence/ Divergence	Integrated Interpretation
Governance frameworks	3.5 (Functional)	Frameworks referenced but applied variably; urgency often overrides written procedures	Federal actors reported more consistent application than woreda-level actors	Convergence	Formal frameworks exist and are widely recognized, but implementation fidelity is inconsistent. The gap between policy and practice widens at subnational levels and during non-emergency periods.
Institutional roles	3.3 (Functional)	Roles defined in policy documents but overlap frequently during emergencies	Agriculture and livestock sectors reported parallel reporting lines as a specific source of confusion	Convergence	Written role clarity degrades under crisis pressure. Overlap is most pronounced during zoonotic events where health, agriculture, and livestock mandates intersect without clear leadership designation.
Coordination mechanisms	3.4 (Functional)	Intensifies during emergencies; relies heavily on informal channels (WhatsApp, phone calls)	Some federal respondents reported scheduled coordination meetings; woreda respondents described reactive, event-driven coordination	Divergence	Coordination is perceived as functional in quantitative scores, but qualitative data reveal heavy reliance on informal, non-accountable channels. Formal coordination platforms are activated mainly during emergencies rather than maintained routinely.
Preparedness oversight, equity, transparency	3.1 (Functional)	Reporting inconsistent across levels; limited feedback from higher levels	Regional respondents noted that supervisory visits occur but recommendations are rarely resourced or followed up	Convergence	Oversight mechanisms exist on paper but are not consistently implemented. The weak feedback loop from higher to lower administrative levels undermines accountability and learning.
Challenges with policy frameworks	3.0 (Emerging)	Limited training, unclear operational guidance, resource constraints	Some federal respondents acknowledged these gaps; woreda respondents described them as pervasive and system-wide	Convergence	This domain scored lowest quantitatively and was described most critically qualitatively. Policy implementation is the weakest area, with respondents consistently citing mismatches between policy requirements and available operational capacity.
Strengthening multisector coordination	3.5 (Functional)	Joint teams formed during outbreaks; One Health platform used	Academic partners reported providing technical input but described their role as ad hoc rather than institutionalized	Expansion	Coordination strengthening occurs mainly during active emergencies. The relatively high quantitative score may reflect recent outbreak experiences, while qualitative data suggest these mechanisms are not yet routine or sustainable between emergencies.
Implementation strategies	3.3 (Functional)	Pragmatic sequencing (risk assessment → task assignment → logistics); frequent adaptation and improvisation	Woreda respondents described borrowing staff and vehicles from other departments as routine practice	Expansion	Implementation is adaptive and context-driven. While this flexibility enables response under resource constraints, the frequency of improvisation signals systemic gaps in institutional support, logistics, and surge capacity.

## 4. Discussions

This study examined multisector emergency governance in Ethiopia using an integrated quantitative and qualitative approach. Overall, governance arrangements were assessed at a functional level, indicating that core frameworks, coordination mechanisms, and implementation structures are formally established. Recognizing that these findings reflect stakeholder perceptions rather than independently verified performance metrics (e.g., after-action review scores or documented reporting timeliness), the following interpretation should be read with appropriate caution. However, performance varied across domains, with policy implementation consistently emerging as the weakest area. While national guidelines and coordination platforms are widely recognized, their application remains uneven across sectors and administrative levels, particularly at subnational levels where governance practices are shaped by resource constraints, contextual pressures, and informal arrangements.

Policy frameworks most clearly illustrate the gap between formal alignment and effective implementation. Although policies are in place, respondents described persistent constraints related to limited training, unclear operational guidance, and insufficient resources, especially at regional and woreda levels. These challenges point not to policy absence, but to difficulties sustaining coherent and coordinated implementation in practice. Similar patterns have been documented across low- and middle-income settings, where well-articulated national emergency policies coexist with weak institutionalisation and uneven subnational uptake, particularly in sub-Saharan Africa and South Asia [24,33,34]. Reviews of public health preparedness further show that policy effectiveness depends less on framework design than on workforce capacity, operational clarity, and implementation support [33,35–37].

Institutional roles and coordination mechanisms displayed a comparable pattern. Although rated as functional overall, qualitative findings indicate that coordination arrangements are frequently strained during active emergencies, with role overlap, parallel reporting, and coordination delays reported as routine. These dynamics are consistent with evidence from major infectious disease emergencies, including Ebola and COVID-19, where emergency pressures amplified role ambiguity and institutional competition despite established coordination structures [24,-38–40]. Recent research on Pakistan’s public health system similarly found that decentralization without clear federal oversight mechanisms fragmented governance, created coordination vacuums, and left national public health agencies under-resourced to serve as effective coordinating authorities [41].

The coordination challenges identified in our study role ambiguity, institutional competition, parallel reporting lines, and delays in information sharing are not unique to Ethiopia. These patterns have been consistently documented during major public health emergencies globally. During the 2014–2015 West African Ebola outbreak, Sierra Leone’s response coordination was hampered by “competition and duplication of efforts between the numerous coordination groups” and a “lack of clarity about the roles, responsibilities, and channels of communication” between national and district-level structures [42]. Similarly, during the COVID-19 pandemic, coordination failures emerged even in high-resource settings. In the United States, overlapping mandates between the Department of Health and Human Services (HHS) and the Federal Emergency Management Agency (FEMA) created competing authorities and confusion over pandemic response roles, with both agencies establishing duplicative supply chains and competing for resources [43,44]. Federal and state officials reported that poor agency communication and unclear roles left the United States without a clear federal pandemic leader during critical early months [43,44]. These international experiences mirror our Ethiopian findings, where participants described overlapping responsibilities during zoonotic events, parallel reporting arrangements between Agriculture and Health sectors, and coordination delays when one sector failed to share timely information. This convergence of evidence across diverse settings suggests that coordination failures during health emergencies may be systemic rather than context-specific, arising from the inherent challenges of rapid multisectoral mobilization under crisis conditions. This suggests that formal role definitions alone are insufficient to ensure effective coordination under crisis conditions.

Preparedness oversight and transparency also functioned unevenly. While reporting and evaluation mechanisms formally exist, respondents described irregular reporting, variable data quality, and limited feedback, particularly outside major emergencies. As a result, oversight mechanisms do not consistently support accountability or institutional learning. This pattern aligns with global evidence highlighting persistent weaknesses in monitoring, feedback, and learning processes in emergency governance systems [45–48], including limited follow-up on Joint External Evaluation findings [48–50].

Implementation practices further revealed how governance systems operate under constraint. Although implementation strategies were rated as functional, respondents described frequent reliance on adaptation, improvisation, and informal resource mobilization. While adaptive capacity is widely recognized as essential in resource-limited settings [51–53], sustained dependence on informal practices may signal gaps in institutional support and long-term implementation planning [54–56]. In this study, adaptation was consistently framed as a response to constraint rather than as a deliberate governance strategy.

Differences across administrative levels emerged as a defining feature of governance performance. Federal-level actors more often described established structures and clearer coordination arrangements, whereas regional and woreda-level respondents emphasized operational constraints and role overlap. Similar vertical gaps have been reported in other decentralized health systems, where formal national compliance coexists with persistent subnational challenges related to financing, workforce capacity, and service delivery [57–61]. Governance literature further cautions that decentralization can exacerbate fragmentation and inequity when intergovernmental coordination and oversight are weak [57–59,62,63].

Taken together, these findings suggest that strengthening emergency governance depends less on introducing new frameworks than on ensuring that existing arrangements function effectively in practice. Improving policy-to-practice translation requires sustained investment in operational guidance, routine training, and subnational implementation support. Regular use of coordination platforms outside crisis periods, combined with joint planning and after-action reviews, is critical for reducing role ambiguity before emergencies occur. From this perspective, adaptive practices should be interpreted as indicators of system stress rather than substitutes for institutionalised governance, underscoring the value of integrating performance scoring with qualitative insight to understand how governance systems function when most tested.

## 5. Strength and limitations

This study integrates quantitative and qualitative methods to examine multisector emergency governance as both a formal institutional arrangement and an operational practice. The high survey response rate across diverse sectors strengthens confidence in the robustness of the quantitative findings, while the qualitative data provide contextual insight into how governance arrangements function in practice. Anchoring the analysis in a governance framework aligned with global standards, while retaining contextual specificity, enhances the relevance of the findings for national policy and comparative research.

Several limitations warrant specific reflection. First, quantitative results are based on self-reported perceptions rather than event-specific independent evaluations. Self-reports may introduce social desirability bias, potentially overestimating governance performance where respondents wish to present their institutions favorably. Second, representation of woreda-level actors was limited (17.6% of quantitative respondents). Because qualitative data consistently showed that frontline implementation challenges role overlap, resource constraints, and reliance on informal coordination were most acute at lower administrative levels, the underrepresentation of woreda voices likely means quantitative domain scores underestimate the severity of implementation gaps. Third, the study sample was predominantly male (79.7%), which limits our ability to examine potential gender-based differences in governance perceptions or experiences. Future studies should intentionally oversample district-level actors and employ purposive gender-stratified sampling to obtain more representative and equitable estimates.

## 6. Recommendations

Strengthening multisector emergency governance in Ethiopia should focus on improving the implementation of existing frameworks rather than introducing new policies or coordination structures. Priority should be given to translating formal multisector commitments into clearly defined, consistently applied operational practices across all levels of the system.

At the federal level, national multisector guidelines should be converted into concise, role-specific operational guidance that clearly differentiates responsibilities by type of public health emergency, including epidemic-prone diseases, zoonotic outbreaks, climate-related shocks, and complex emergencies. This guidance should explicitly specify leadership, coordination, decision-making, and accountability functions across preparedness, response, and recovery phases. Practice-oriented training for sectoral focal points should accompany this process, focusing on applied decision-making, coordination interfaces, and accountability under realistic emergency conditions, informed by recent outbreak experiences such as Marburg.

Roles and coordination arrangements across sectors should be routinely tested and refined through structured, scenario-based multisector workshops conducted outside crisis periods. These exercises should simulate different emergency types and response trajectories to identify coordination bottlenecks, clarify handover points, and reinforce shared expectations regarding leadership and information flow.

At regional and woreda levels, sustained implementation support is required to address recurring coordination challenges, role overlap, and reliance on informal arrangements. Regional authorities should routinely convene multisector coordination platforms during non-emergency periods to clarify reporting lines, decision pathways, and institutional responsibilities. Targeted subnational support, including protected staff time and limited operational resources, should be prioritised to address capacity constraints that undermine consistent governance practice.

Preparedness oversight should be strengthened through regular review of preparedness and response reports, with documented follow-up actions, assigned responsibilities, and timelines. After-action and real-time reviews following major public health emergencies, including events such as the Marburg outbreak, should be institutionalised as a core governance function. Findings from these reviews should be systematically integrated into operational guidance, standard operating procedures, and capacity-building efforts.

Finally, adaptive coordination practices that emerge at subnational levels should be formally documented and assessed to inform gradual strengthening of more standardised governance processes. Multisector collaboration should be reinforced not only during acute response phases but also across preparedness and recovery through routine joint planning, shared information systems, and clearly defined cross-sector accountability mechanisms.

## 7. Conclusions

Within the limitations of self-reported perception data, this study demonstrates that multisector emergency governance in Ethiopia is supported by established policy and coordination frameworks that provide a functional institutional foundation, yet their implementation varies substantially in practice. Quantitative findings indicate moderate overall functionality across governance domains, while qualitative evidence shows that the application of policies, coordination arrangements, and accountability mechanisms during emergencies often depends on adaptive practices, informal coordination, and individual initiative, particularly at subnational levels. These findings indicate that the functioning of multisector emergency governance cannot be understood through the presence of formal structures alone, but must be examined in relation to how governance arrangements are operationalized under real-world conditions. By integrating quantitative and qualitative evidence, this study provides a clearer understanding of how multisector emergency governance is applied in practice and identifies persistent gaps in policy implementation, coordination, and oversight that warrant sustained attention across administrative levels.

## Supporting information

S1 File Survey Questionnaire and Key Informant Interview Guide. This file contains the complete quantitative survey instrument (Section A: demographic and organizational practices; Section B: 5-point Likert items across seven governance domains) and the semi-structured qualitative interview guide aligned with the same seven domains.
(DOCX)

S1 Table Detailed participant-level qualitative findings and illustrative quotations by governance domain. This table provides a comprehensive domain-by-domain synthesis of qualitative data, including representative verbatim quotations from key informants, organized by governance domain and sub-theme. Quotations are anonymized and attributed by participant role and administrative level.
(DOCX)

## Abbreviations

DRM: Disaster Risk Management
EPHI: Ethiopian Public Health Institute
IHR: International Health Regulations
IMS: Incident Management System
JEE: Joint External Evaluation
LMICs: Low- and middle-income countries
NGO: Non-governmental organization
PHEM: Public Health Emergency Management
PHEOC: Public Health Emergency Operations Center
SPAR: State Party Annual Reporting
SPSS: Statistical Package for the Social Sciences
WHO: World Health Organization
