# Supplementary material for: Assessing the policy and institutional framework for multisectoral governance and accountability in emergency preparedness and response in Ethiopia
Source: PLOS Glob Public Health. 2026 Jul 24;6(7):e0006915. doi: 10.1371/journal.pgph.0006915 (PMC13399327; doi:10.1371/journal.pgph.0006915)
Supplement: S1 File — This file contains the complete quantitative survey instrument (Section A: demographic and organizational practices; Section B: 5-point Likert items across seven governance domains) and the semi-structured qualitative interview guide aligned with the same seven domains. (DOCX) [file pgph.0006915.s001.docx]

**S1 File: Assessment tool**

# **Section A. Stakeholder Demographics & Institutional Context**

| # | Question | Response Options |
| --- | --- | --- |
| 1 | How old are you? | _______________years |
| 2 | Sex of the respondent | Male ………………………………….1  Female………………………………...2 |
| 3 | Educational level | Diploma……………………………….1  Degree (BSc/BA)……………………...2  MD/DVM ………………………….….3  Masters(MSc/MPH/MA)…………..….4  PhD………………………………....….5 |
| 4 | What type of institution do you represent? | Federal ministry/agency………………1  Regional health bureau………..………2  NGO………………………………..…3  Academia…………………………..…4  Other (Specify)______________ ……5 |
| 5 | Which primary sector best describes your work? | Health…………………………………1  Disaster risk management…….………2  Agriculture…………………....………3  Education……………………..………4  Other (Specify)______________ ……5 |
| 6 | At what administrative level does your organization operate? | National………………………………1  Regional………………………...……2  Woreda…………………………….…3 |
| 7 | What is your primary role in emergency governance? | Planner……………………….………1  Technical lead………….…….………2  Coordinator………………….….……3  Expert………………………………..4  Other (Specify)______________ …...5 |
| 8 | How many years of experience do you have in Public Health Emergency Management (PHEM)? | ___________years |
| 9 | How familiar are you with governance frameworks (e.g., IHR, Sendai)? | Not aware………………………...…1  Somewhat aware………………....…2  Fully aware…………………….....…2 |
| 10 | How familiar are you with the national Incident Management System (IMS)? | Not aware………………………...…1  Somewhat aware….……………...…2  Fully aware……………………….....3 |
| 11 | Have you attended a combined-hazard management workshop in the past 2 years (e.g., covering disease outbreaks, floods, droughts)? | No………………………...............…0  Yes……………………………......…1 |
| 12 | Were you deployed to an emergency response in the last 12 months? | No………………………...............…0  Yes……………………………......…1 |
| 13 | How would you rate your experience with risk-profiling tools (Hot spot classification/ STAR/WDRP)? | Not aware………………………...….1  Heard of………………………...…...2  Have used………………………...….3 |
| 14 | Does your organization maintain a dedicated emergency coordination unit? | No………………………...............…0  Yes……………………………......…1 |
| 15 | Do you have access to emergency policy documents? | No………………………...............…0  Yes……………………………......…1 |
| 16 | Do you use digital platforms for PHEM coordination? | No………………………...............…0  Yes……………………………......…1 |
| 17 | How frequently does your organization convene inter-sector meetings? | Never………………………...………1  Occasionally………………………....2  Frequently………………………...….3 |
| 18 | Do you routinely apply SOPs or legal mandates in PHEM activities? | No………………………...............…0  Yes……………………………......…1 |
| 19 | How would you rate political support for PHEM initiatives? | Low………………………...………...1  Moderate………………………......…2  High……………………….............…3 |
| 20 | How equitable is resource allocation among sectors? | Not equitable………………………...1  Partially equitable………………....…2  Fully equitable……………………….3 |
| 21 | How would you rate trust in inter-agency collaboration? | Low………………………………..….1  Moderate…………………………...…2  High………………………..............…3 |
| 22 | Does your organization have a structured community-engagement mechanism? | No………………………...............…0  Yes……………………………......…1 |
| 23 | Are external accountability reviews conducted within your organization? | No………………………...............…0  Yes……………………………......…1 |

# **Section B. Stakeholder** **Perception Questionnaire (5‑Point Likert)**

Circle one number per item where:
1 = Strongly Disagree  2 =  Disagree 3 =  Neutral 4 =  Agree 5 = Strongly Agree

## Governance Frameworks

| Item # | Statement | Strongly Disagree | Disagree | Neutral | Agree | Strongly Agree |
| --- | --- | --- | --- | --- | --- | --- |
| 1 | Current policy guidelines clearly define emergency roles and responsibilities. | 1 | 2 | 3 | 4 | 5 |
| 2 | Policy guidelines are accessible to all relevant stakeholders. | 1 | 2 | 3 | 4 | 5 |
| 3 | Policy guidelines are regularly reviewed to reflect best practices. | 1 | 2 | 3 | 4 | 5 |
| 4 | Stakeholders receive training on implementing policy guidelines. | 1 | 2 | 3 | 4 | 5 |

## Institutional Role

| Item # | Statement | Strongly Disagree | Disagree | Neutral | Agree | Strongly Agree |
| --- | --- | --- | --- | --- | --- | --- |
| 5 | Agency roles in emergency management are clearly defined. | 1 | 2 | 3 | 4 | 5 |
| 6 | All stakeholders understand their roles in preparedness and response. | 1 | 2 | 3 | 4 | 5 |
| 7 | Capacity‑building programmes are provided regularly. | 1 | 2 | 3 | 4 | 5 |
| 8 | Emergency management agencies have sufficient authority. | 1 | 2 | 3 | 4 | 5 |

## Coordination Mechanisms

| Item # | Statement | Strongly Disagree | Disagree | Neutral | Agree | Strongly Agree |
| --- | --- | --- | --- | --- | --- | --- |
| 9 | Multi‑sectoral coordination bodies are established. | 1 | 2 | 3 | 4 | 5 |
| 10 | Effective communication frameworks exist for information sharing. | 1 | 2 | 3 | 4 | 5 |
| 11 | Stakeholders collaborate effectively to mobilise resources. | 1 | 2 | 3 | 4 | 5 |
| 12 | Coordination bodies meet regularly on emergencies. | 1 | 2 | 3 | 4 | 5 |

## Preparedness Oversight, Equity, and Transparency

| Item # | Statement | Strongly Disagree | Disagree | Neutral | Agree | Strongly Agree |
| --- | --- | --- | --- | --- | --- | --- |
| 13 | Regular evaluations identify weaknesses in preparedness policies. | 1 | 2 | 3 | 4 | 5 |
| 14 | Local stakeholders are actively engaged in decision‑making. | 1 | 2 | 3 | 4 | 5 |
| 15 | Resources are allocated equitably among communities. | 1 | 2 | 3 | 4 | 5 |
| 16 | Stakeholders are informed about resource allocation decisions. | 1 | 2 | 3 | 4 | 5 |

## Challenges with Policy Frameworks

| Item # | Statement | Strongly Disagree | Disagree | Neutral | Agree | Strongly Agree |
| --- | --- | --- | --- | --- | --- | --- |
| 17 | Strategies ensure compliance with preparedness laws. | 1 | 2 | 3 | 4 | 5 |
| 18 | Political leaders show commitment to preparedness. | 1 | 2 | 3 | 4 | 5 |
| 19 | Sustainable funding mechanisms exist for preparedness. | 1 | 2 | 3 | 4 | 5 |
| 20 | Bureaucratic processes do not hinder response efforts. | 1 | 2 | 3 | 4 | 5 |

## Strengthening Multi‑Sector Coordination

| Item # | Statement | Strongly Disagree | Disagree | Neutral | Agree | Strongly Agree |
| --- | --- | --- | --- | --- | --- | --- |
| 21 | Institutional reforms are recommended based on assessments. | 1 | 2 | 3 | 4 | 5 |
| 22 | Community members are actively involved in planning. | 1 | 2 | 3 | 4 | 5 |
| 23 | Innovative technologies enhance coordination. | 1 | 2 | 3 | 4 | 5 |
| 24 | Feedback mechanisms gather stakeholder input. | 1 | 2 | 3 | 4 | 5 |

## Implementation Strategy

| Item # | Statement | Strongly Disagree | Disagree | Neutral | Agree | Strongly Agree |
| --- | --- | --- | --- | --- | --- | --- |
| 25 | Evidence‑based practices are implemented. | 1 | 2 | 3 | 4 | 5 |
| 26 | Stakeholders are engaged throughout implementation. | 1 | 2 | 3 | 4 | 5 |
| 27 | Strategies adapt to local context for relevance. | 1 | 2 | 3 | 4 | 5 |
| 28 | Clear metrics monitor and evaluate implementation. | 1 | 2 | 3 | 4 | 5 |
| 29 | A phased approach is used for implementation. | 1 | 2 | 3 | 4 | 5 |
| 30 | Effectiveness of evidence‑based practices is assessed. | 1 | 2 | 3 | 4 | 5 |

**Part 2: Qualitative Component**

**Key Informant Interview / In-Depth Interview Guide**

Participant: Targeted at individuals with specific knowledge of *emergency preparedness and response* programs and related service or program delivery.

**Multisector Governance and Accountability in Health Emergency Preparedness**

This guide provides a structured approach to gather qualitative insights across seven governance domains. It is designed for use in interviews, focus group discussions (FGDs), or key informant interviews to support comprehensive evaluations of emergency preparedness systems.

**Purpose**

To explore institutional practices, barriers, enablers, and perceptions across governance domains to complement quantitative scoring frameworks and inform actionable improvements.

Qualitative Interview Guide – Governance Domains and Prompts

KII Guide (45–60 minutes)

**Instructions for Interviewer:**

- Cover **3–4 domains** per interview.
- Ask all three questions per domain, then use probes as needed.
- Conclude by asking for top priorities across domains.

**Introduction**
Thank you for your time. This study aims to explore Ethiopia’s policy and institutional frameworks for multisectoral governance and accountability in emergency preparedness and response. The primary objective is to identify systemic gaps, stakeholder perceptions, and best practices that can inform a robust, context-specific governance model. The study also seeks to develop evidence-based recommendations to enhance institutional coordination, legal mandates, and stakeholder engagement in managing public health emergencies. Your input is valuable for understanding the effectiveness and challenges of these programs.

**Background Information**

| Characteristics | Response | Remarks |
| --- | --- | --- |
| Age | _________years |  |
| Sex | 1. Male 2. Female |  |
| Educational level | ________________________ |  |
| Name of Organization (don’t ask just fill it) | ________________________ |  |
| Position (current) | ________________________ |  |
| Years of experience | ____________years |  |

| Domain | Core Questions |
| --- | --- |
| **1. Governance Frameworks** | 1. “Please describe which governance structures or processes have most effectively supported multisector PHEM.” 2. “What specific obstacles have hindered full implementation of these frameworks?” 3. “What changes would strengthen them?” |
| **2. Institutional Roles** | 1. “How are roles and responsibilities currently defined among institutions involved in PHEM?” 2. “Can you share an example of where role overlap or gaps occurred?” 3. “What mechanisms could improve role clarity?” |
| **3. Coordination Mechanisms** | 1. “Walk me through how information is shared across sectors during an emergency.” 2. “What barriers prevent smoother coordination?” 3. “Which platforms or practices would you recommend to enhance coordination?” |
| **4. Resource Equity & Trust** | 1. “Tell me how resources (funding, personnel, equipment) are allocated across sectors.” 2. “What factors drive perceived inequities or erode trust?” 3. “What policies or processes could improve equity and trust?” |
| **5. Data & Information Systems** | 1. “Describe the tools and platforms you use for emergency data collection and analysis.” 2. “What technical or operational constraints limit their effectiveness?” 3. “How could data systems be optimized for PHEM?” |
| **6. Community Engagement & Accountability** | 1. “How are communities involved in planning and evaluating responses?” 2. “What barriers prevent meaningful community participation?” 3. “Which engagement practices have worked well, and why?” |
| **7. Workforce Capacity** | 1. “Explain how training and support are delivered to frontline responders.” 2. “What workforce challenges (e.g., retention, skills gaps) most affect performance?” 3. “What interventions could bolster workforce capacity?” |

**Probes:**

- “Can you illustrate with a recent example?”
- “Why do you think that issue persists?”
- “What resources or policy changes are needed?”

**Closing (all domains):**
“Of all the enablers, barriers, and opportunities discussed, which single factor should be prioritized for immediate action, and why?”
