# Supplementary material for: Assessing the policy and institutional framework for multisectoral governance and accountability in emergency preparedness and response in Ethiopia
Source: PLOS Glob Public Health. 2026 Jul 24;6(7):e0006915. doi: 10.1371/journal.pgph.0006915 (PMC13399327; doi:10.1371/journal.pgph.0006915)
Supplement: S1 Table — This table provides a comprehensive domain-by-domain synthesis of qualitative data, including representative verbatim quotations from key informants, organized by governance domain and sub-theme. Quotations are anonymized and attributed by participant role and administrative level. (DOCX) [file pgph.0006915.s002.docx]

S1 Table: Participant-Level Summary of Pertinent Qualitative Findings Across All Interview Domains

| Participant ID | Institution / Sector | Position | Years of Experience | Pertinent Findings | Illustrative Quotes |
| --- | --- | --- | --- | --- | --- |
| P1 | Ethiopian Public Health Institute (EPHI) | PHEM expert | 11 | The participant explained that multisector coordination becomes structured and functional mainly when a formal emergency is declared and the PHEOC is activated. During these periods, roles, reporting lines, and decision-making processes are clear. Outside active emergencies, however, coordination weakens considerably and depends largely on individual initiative. The effectiveness of governance structures was described as person-dependent rather than institutionalized, making continuity fragile when experienced leaders are transferred or replaced. | “Once the PHEOC is activated, everything becomes organized.”  “During emergencies IMS really works, but outside that time coordination is very loose.”  “If certain people leave, the system becomes weak again.” |
| P2 | EPHI | PHEM officer | 8 | This participant described a system where institutional roles are formally defined but unevenly understood, particularly at regional and woreda levels. As a result, institutions often respond in parallel rather than jointly, especially during zoonotic events. Surveillance relies heavily on informal communication for speed, with official systems often lagging behind. This creates duplication, delays, and confusion at community level, even though individual responders act with good intentions. | “On paper roles are clear, but in practice it becomes messy.”  “Both sectors sometimes investigate the same outbreak separately.”  “Many alerts reach us first through WhatsApp, not official reports.” |
| P3 | EDRMC | Senior Early Warning Officer | 13 | The participant highlighted that Ethiopia’s disaster risk management system benefits from long experience with recurrent crises, which has built a strong coordination culture within DRM structures. However, these systems do not consistently integrate with public health emergency mechanisms. Health and DRM often operate side by side rather than as a single system, unless senior leadership actively enforces collaboration. Frequent leadership changes and weak legal mandates further undermine sustained integration. | “DRM coordination works well because we have done this for years.”  “Health and DRM often activate separately.”  “Without enforcement, coordination depends on personalities.” |
| P4 | EDRMC | Senior Early Warning Analyst | 10 | This participant emphasized that the national early warning bulletin is one of the few platforms that genuinely brings multiple sectors together around a shared risk picture. Despite this strength, the system is constrained by inconsistent reporting from regions, limited analytical capacity, and political sensitivity around reporting worsening conditions. Public health surveillance data are not systematically embedded in early warning analysis, reducing the system’s ability to anticipate outbreaks. | “The bulletin forces everyone to look at the same picture.”  “Some regions hesitate to report worsening conditions.”  “Health data often arrive too late for prediction.” |
| P5 | Ministry of Agriculture (MoA) | Senior Veterinary Public Health Officer | 12 | The participant described agriculture as being consistently engaged late in emergency response, even when outbreaks originate in animals. Although One Health structures exist, limited financing, outdated joint guidelines, and overstretched veterinary services prevent effective implementation. Animal health preparedness was described as secondary to human health priorities, despite its critical role in preventing zoonotic spillover. | “Most outbreaks start in animals, but we hear about them after human cases.”  “Guidelines exist, but they are not practical on the ground.”  “Without budget, coordination stays on paper.” |
| P6 | Regional Bureau of Agriculture | Regional Animal Health Officer | 9 | At regional level, the participant explained that coordination depends more on informal relationships than formal frameworks. National policies rarely reach frontline workers, and implementation is shaped by resource shortages, political pressure, and local leadership attitudes. Even when staff understand what should be done, lack of fuel, transport, and authority limits their ability to act quickly. | “Governance works through relationships, not documents.”  “Most national strategies never reach woreda staff.”  “Sometimes reporting is delayed to avoid attention.” |
| P7 | Federal Veterinary Services | Veterinary Epidemiologist | 14 | This participant described a fragmented system in which sectoral mandates, slow approval processes, and unaligned reporting pathways delay outbreak investigation and confirmation. Informal communication helps compensate for these weaknesses but also creates risks, as information can be lost or fail to reach all relevant actors. The system’s effectiveness was described as highly dependent on who is on duty at a given time. | “Emergencies don’t wait for approvals.”  “We often chase the same outbreak from two directions.”  “If we relied only on letters, we would always be late.” |
| P8 | FAO Ethiopia | Emergency & Resilience Officer | 15 | From a partner perspective, the participant observed that trust-based informal coordination enables rapid action and early warning, particularly in animal health emergencies. However, fragmented systems, limited resources, and multiple parallel partner tools increase the burden on government counterparts and complicate information management. Informal coordination was seen as effective but unsustainable without institutional backing. | “One phone call can mobilize action faster than any memo.”  “Everyone brings tools, and the system becomes crowded.”  “Without resources, frameworks don’t function.” |
| P9 | EPHI | PHEM expert | 10 | The participant explained that preparedness structures exist but receive less attention, funding, and recognition than response activities. Sectoral silos, lack of shared data platforms, and high staff turnover weaken sustained preparedness efforts. Preparedness was described as undervalued because its success is difficult to see when crises are prevented rather than responded to. | “Preparedness feels optional because its success is invisible.”  “We pull data from many systems to see the full picture.”  “Turnover resets coordination again and again.” |
| P10 | Federal Defense Health Division | PHEM expert | 16 | This participant highlighted the strong logistical, medical, and organizational capacity within the defense sector, noting that it is often underutilized during preparedness phases. Differences in institutional culture and late engagement limit effective civil–military integration. Earlier and clearer inclusion of defense health capacities was seen as essential for national readiness, particularly in remote and insecure areas. | “We are often engaged only when risk is already high.”  “Military systems move fast; civilian systems take longer.”  “Earlier integration would strengthen preparedness.” |
