# Supplementary material for: Assessing the policy and institutional framework for multisectoral governance and accountability in emergency preparedness and response in Ethiopia
Source: PLOS Glob Public Health. 2026 Jul 24;6(7):e0006915. doi: 10.1371/journal.pgph.0006915 (PMC13399327; doi:10.1371/journal.pgph.0006915)
Supplement: Attachment — Submitted filename: Point by point response.docx [file pgph.0006915.s004.docx]

**RESPONSE TO REVIEWERS**

**Manuscript ID:** PGPH-D-26-00736

**Title:** Assessing the Policy and Institutional Framework for Multisectoral Governance and Accountability in Emergency Preparedness and Response in Ethiopia

**Journal:** PLOS Global Public Health

**Corresponding Author:** Sileshi Demelash Sasie

**Email:** sileyeshi21@gmail.com
**ORCID:** 0000-0002-7273-8923

**Date of Submission:** 23/5/2026

**Dear Dr. Robinson and Distinguished Reviewers,**

On behalf of my co-authors, I extend our sincere gratitude for the thoughtful, rigorous, and constructive reviews of our manuscript. The editorial team and reviewers have provided exceptional guidance that has materially improved the scientific clarity, methodological transparency, and interpretative caution of our work.

We have carefully addressed each comment. Below, we present the editor's requirements and each reviewer's critique, followed by our detailed response and the specific revisions made. All page and line numbers refer to the clean, revised version of the manuscript.

**EDITORIAL COMMENTS**

**Comment E1: Ethics Statement – Verbal Consent Documentation**

In the ethics statement in the Methods, you have specified that verbal consent was obtained. Please provide additional details regarding how this consent was documented and witnessed, and state whether this was approved by the IRB.

**Response:** We acknowledge the need for procedural specificity. The verbal consent process was explicitly approved by the Hawassa University IRB, and we have now detailed the documentation and witnessing procedures.

**Revision:** Ethical approval for this study was obtained from the Hawassa University Institutional Review Board (IRB/536/17; June 30, 2025). Verbal informed consent was obtained from all participants prior to enrollment. For in-person interviews, consent was documented by the data collector using a pre-printed consent log, which recorded the participant’s name, date, and confirmation that consent was freely given. An independent witness (a second data collector not involved in administering that interview) was present during the consent process to verify that information was fully disclosed and that consent was voluntary.

For online survey participants, consent was documented via a required electronic check-box acknowledgement before survey access. This electronic procedure was specifically approved by the IRB as equivalent to written consent. Participation was voluntary, no personally identifiable information was collected, and all data were anonymized and stored securely in password-protected files accessible only to the research team.

**Location:** Methods / Ethics Statement (lines 219-229)

**Comment E2: Data Availability – Restrictions and Access**

Please explain legal/ethical restrictions on data sharing and provide contact information for a data access committee.

**Response:** We have deposited de-identified quantitative data in a public repository and provided a clear access pathway for qualitative data under IRB oversight.

**Revision:** De-identified quantitative data supporting the findings of this study are publicly available in the Zenodo repository: Sasie, S. D. (2026). Assessing the Policy and Institutional Framework for Multisectoral Governance and Accountability in Emergency Preparedness and Response in Ethiopia [Data set]. Zenodo. <https://doi.org/10.5281/zenodo.20351876>.

Qualitative data (key informant interview transcripts) are not publicly available due to ethical restrictions imposed by the Hawassa University Institutional Review Board (IRB/536/17), as transcripts contain potentially identifying institutional and role-specific information. Requests for de-identified qualitative data excerpts may be directed to the corresponding author (Sileshi Demelash Sasie, sileyeshi21@gmail.com). All requests will be reviewed by the Hawassa University IRB (irb@hu.edu.et) to ensure compliance with ethical and confidentiality guidelines.

**Location:** Declarations / Availability of Data and Materials (lines 582-591)

**Comment E3: Ethics Statement Placement**

Please ensure that your Ethics Statement is available in its entirety at the beginning of your Methods section, under a subheading 'Ethics Statement.

**Response:** We have relocated the full ethics statement as requested.

**Revision:**

- A new subheading “Ethics Statement” has been created at the beginning of the Methods section (immediately after the study design paragraph).
- The complete ethics text (as revised in response E1) now appears there.
- The previous abbreviated ethics statement in the Declarations section has been removed to avoid duplication.

**Location:** Methods / Ethics Statement (lines 217-229)

**Comment E4: Supporting Information Legends**

*Please add a full list of legends for your Supporting Information files after the references list.*

**Response:** We have added a complete, descriptive list of legends.

**Revision:**

**Supporting Information Legends**

**S1 File. Survey Questionnaire and Key Informant Interview Guide.** Quantitative survey (demographics + 30 Likert items across 7 governance domains) and qualitative key informant interview guide.

**S1 Table. Participant-Level Summary of Qualitative Findings.** Participant-level qualitative synthesis from 10 key informants, including findings, quotes, and institutional context.

**Location:** After References (lines 799-809)

**REVIEWER #1 COMMENTS**

**Comment R1-1: Maturity Framework – Conceptual Overclaim**

*The ‘maturity’ scoring risks appearing arbitrary. The categories are described as ‘a priori’ but referenced to an unpublished national framework, which is insufficient. Ground cutoffs in published precedent or re-frame as descriptive averages without maturity labeling.*

**Response:** Thank you so much for these constructive comments. We agree that presenting the maturity categories as definitive, validated performance standards was overreaching. We have reframed the maturity classification as descriptive categories, grounded the cutoffs in published literature, and softened all interpretive language. The word "heuristic" has been removed entirely.

**Revisions: Methods**: Scores ranged from 1 to 5 and were categorized into four descriptive maturity levels: nascent (1.0–1.9), emerging (2.0–2.9), functional (3.0–3.5), and optimized (3.6–5.0). These categories were applied as a descriptive analytical device to facilitate comparison across governance domains, not as an externally validated performance standard. The cutoff thresholds are consistent with maturity models used in health systems evaluation[1, 2].

**Results (Table 3)** – Column header changed from "Interpretation" to **“**Descriptive Maturity Level**”**

**Abstract** – Softened claim:

**Original**: The mean overall institutional governance maturity score was 3.31, corresponding to a functional level of maturity.

**Revised:** The mean overall perceived governance maturity score was 3.31, falling within the study’s predefined ‘functional’ descriptive category (3.0–3.5).

**Location:** Methods (lines 193-198); Results / Table 3; Abstract (lines 37-39).

**Comment R1-2: Woreda Representation**

Only 13 of 74 respondents (17.6%) were from ‘woreda’ (district) level, yet qualitative findings repeatedly claim subnational governance is weakest. State more directly that quantitative scores are not reliably representative of frontline perspectives.

**Response:** We agree entirely. The quantitative scores disproportionately reflect federal and regional views. We have stated this limitation directly and explicitly.

**Revision:** Second, representation of woreda-level actors was limited (17.6% of quantitative respondents). Because qualitative data consistently showed that frontline implementation challenges role overlap, resource constraints, and reliance on informal coordination were most acute at lower administrative levels, the underrepresentation of woreda voices likely means quantitative domain scores underestimate the severity of implementation gaps. Third, the study sample was predominantly male (79.7%), which limits our ability to examine potential gender-based differences in governance perceptions or experiences. Future studies should intentionally oversample district-level actors and employ purposive gender-stratified sampling to obtain more representative and equitable estimates.

**Location:** Limitations (lines 514-522)

**Comment R1-3a: Mixed-Methods Integration – Joint Display**

There is no systematic ‘joint display’ or cross-tabulation showing how respondents who rated ‘policy frameworks’ as emerging differed in their qualitative accounts. Without this, the claim of ‘sequential mixed-methods integration’ is not fully realized.

**Response:** We thank the reviewer for this important methodological critique. We agree that a formal joint display is essential to demonstrating genuine mixed-methods integration rather than simply presenting quantitative and qualitative findings in parallel. To address this, we have constructed a comprehensive integrated matrix that systematically aligns quantitative domain scores with corresponding qualitative themes and provides a synthesized interpretation for each domain.

**Methodological approach to integration:** Following established mixed-methods procedures (Fetters, Curry, & Creswell, 2013), we used a joint display framework where quantitative domain-level mean scores were first calculated, then qualitative data were coded and thematically analyzed independently, and finally both streams were integrated at the interpretation stage through side-by-side comparison. For each of the seven governance domains, we identified convergence (where quantitative and qualitative findings pointed in the same direction), divergence (where they appeared contradictory), and expansion (where qualitative findings provided explanatory depth beyond the quantitative score). This process is summarized in the matrix below.

**Revision:**

**3.6 Integrated Mixed-Methods Matrix Across Governance Domains**

Table 5 integrates the quantitative and qualitative findings. Quantitative scores are means on a 1–5 scale, with descriptive categories in parentheses. Qualitative dominant themes represent the most frequently reported patterns. Dissenting views capture variation by administrative level or sector. Convergence, divergence, and expansion are defined in the Methods.

Table 5. Integrated Mixed-Methods Matrix: Quantitative Scores, Qualitative Themes, and Synthesized Interpretation Across Governance Domains

**Methods:** Integration of quantitative and qualitative findings was conducted using a joint display matrix. Following established mixed-methods procedures, domain-level quantitative mean scores were compared with qualitatively derived themes. This comparison was structured to assess three types of relationship: convergence (qualitative findings confirming quantitative patterns), divergence (qualitative findings contradicting quantitative patterns), and expansion (qualitative findings providing explanatory depth beyond numerical scores).

**Location:** Results / new Table 5 (line 414-421) ; Methods (lines 208-213)

**Comment R1-3b: Perception vs. Objective Measurement**

All quantitative measures are Likert-scale perceptions of governance, not independent assessments. Draw conclusions about ‘perceived’ governance maturity, not objective performance.

**Response:** This is a critical distinction. We have systematically revised language throughout the manuscript to specify that findings reflect *perceived* governance maturity based on stakeholder self-reports.

**Revisions:**

- **Results section title (3.2):** Changed to **“**Quantitative Domain-Level Perceived Governance Assessment**”**
- **Abstract:** Changed to perceived governance maturity, based on stakeholder self-reports, fell within the study’s predefined ‘functional’ descriptive category
- **Results (paragraph before Table 3):** Changed to *“*mean overall perceived governance maturity score*”*
- **Discussion (first paragraph):** Added *“Recognizing that these findings reflect stakeholder perceptions rather than independently verified performance metrics (e.g., after-action review scores or documented reporting timeliness), the following interpretation should be read with appropriate caution.”*
- **Conclusions:** Added *“within the limitations of self-reported perception data”*

**Location:** Abstract (lines 37-39); Results (line 273); Discussion (lines 426-429); Conclusions (line 558)

**Comment R1-3c: Gender Distribution – Reflexivity**

Gender distribution (79.7% male) is striking but not discussed. A sentence on potential gender bias in governance perceptions or inclusion would strengthen reflexivity.

**Response:** We have added a dedicated reflexive statement.

**Revision:** Third, the study sample was predominantly male (79.7%), which limits our ability to examine potential gender-based differences in governance perceptions or experiences. Future studies should intentionally oversample district-level actors and employ purposive gender-stratified sampling to obtain more representative and equitable estimates.

**Location:** Limitations (lines 518-521)

**Comment R1-3d: Comparison to Ebola and COVID-19**

Comparison to Ebola and COVID-19 coordination challenges is helpful but very brief. Expand by one sentence specifying which coordination failures mirror the Ethiopian findings.

**Response:** We have expanded this comparison with specific parallels.

**Revision:** The coordination challenges identified in our study role ambiguity, institutional competition, parallel reporting lines, and delays in information sharing are not unique to Ethiopia. These patterns have been consistently documented during major public health emergencies globally. During the 2014–2015 West African Ebola outbreak, Sierra Leone's response coordination was hampered by "competition and duplication of efforts between the numerous coordination groups" and a "lack of clarity about the roles, responsibilities, and channels of communication" between national and district-level structures[3]. Similarly, during the COVID-19 pandemic, coordination failures emerged even in high-resource settings. In the United States, overlapping mandates between the Department of Health and Human Services (HHS) and the Federal Emergency Management Agency (FEMA) created competing authorities and confusion over pandemic response roles, with both agencies establishing duplicative supply chains and competing for resources[4, 5]. Federal and state officials reported that poor agency communication and unclear roles left the United States without a clear federal pandemic leader during critical early months[4, 5]. These international experiences mirror our Ethiopian findings, where participants described overlapping responsibilities during zoonotic events, parallel reporting arrangements between Agriculture and Health sectors, and coordination delays when one sector failed to share timely information. This convergence of evidence across diverse settings suggests that coordination failures during health emergencies may be systemic rather than context-specific, arising from the inherent challenges of rapid multisectoral mobilization under crisis conditions. This suggests that formal role definitions alone are insufficient to ensure effective coordination under crisis conditions.

**Location:** Discussion (lines 450-470)

**Comment R1-4: References – Formatting and Missing Citation**

Several citations are incomplete or need formatting. There is a recent paper published in the same journal which could be an interesting reference to cite (PMID: 41920850).

**Response:** We have corrected all reference formatting and added the suggested citation.

**Revisions:**

- All references have been reformatted according to PLOS Global Public Health style (journal names abbreviated consistently, DOIs added).
- The suggested reference (PMID: 41920850) has been added as reference [41] in the discussion section

**Location:** References (lines 650-811); Discussion (line 450-453)

**REVIEWER #2 COMMENTS (Dr. Abiti A.)**

**Comment R2-1: Background – Specific Gap Statement**

More clearly state the specific gap this study is addressing, particularly in terms of what is not yet understood about how governance structures function operationally, beyond what is already described in policies.

**Response:** We have sharpened the gap statement to specify the operational and empirical evidence gap.

**Revision:** Critically, this study addresses a specific evidence gap: while Ethiopia has established policy frameworks (PHEM guidelines, IMS, One Health platform) and coordination structures, very little empirical research has examined how these governance arrangements function operationally particularly the gap between formal policy design and real-world implementation practices across administrative levels, and how this gap varies during and between emergencies.

**Location:** Background (lines 121-125)

**Comment R2-2: Methods – Domain Definition and Indicator Assignment**

Clarify how the seven governance domains were defined and how the indicators were assigned to each domain.

**Response:** We have added a detailed operationalization paragraph.

**Revision:** The seven governance domains were defined a priori based on key principles from the Multisector Emergency Governance Assessment Toolkit and the Guided Assessment for Multisector Governance in Public Health Emergencies. Each domain was operationalized using 4–6 Likert-scale items. For example: the ‘Governance frameworks’ domain included items on clarity of national PHEM guidelines, accessibility of policy documents, and alignment with IHR; the ‘Institutional roles’ domain included items on role clarity across sectors, separation of mandates, and clarity of accountability assignments during emergencies; the ‘Coordination mechanisms’ domain included items on frequency of multisector meetings, clarity of reporting pathways, and use of coordination platforms. A complete domain-item mapping matrix is provided in S1 File.

**Location:** Methods (lines 161-169)

**Comment R2-3: Results – Summary Paragraph**

Add a brief summary paragraph at the beginning of the Results section to highlight the main patterns before moving into the domain-by-domain findings.

**Response:** We have added an orienting summary paragraph.

**Revision:** In brief, three main patterns emerged from the integrated analysis. First, quantitative perceived governance scores across the seven domains ranged from 3.0 to 3.5, with the overall mean perceived maturity score of 3.31 falling within the study’s predefined ‘functional’ descriptive category (3.0–3.5). No domain reached the ‘optimized’ threshold (≥3.6), and the domain addressing ‘challenges with policy frameworks’ scored at the ‘emerging’ level (3.0). Second, qualitative findings revealed substantial variation in how formal frameworks were applied across administrative levels; subnational actors consistently reported greater reliance on informal practices, role overlap, and resource constraints compared with federal respondents. Third, policy implementation emerged as the weakest area across both quantitative and qualitative data, with participants citing limited training exposure, unclear operational guidance, and gaps between policy requirements and available capacity as persistent barriers.

**Location:** Results (lines 263-272)

**Comment R2-4: Limitations – Influence of Self-Report and Woreda Representation**

Reflect on how reliance on self-reported data and limited representation at woreda level might influence the findings, especially in relation to actual implementation at frontline level.

**Response:** We have expanded the limitations section with specific reflection on both issues.

**Revision:** Several limitations warrant specific reflection. First, quantitative results are based on self-reported perceptions rather than event-specific independent evaluations. Self-reports may introduce social desirability bias, potentially overestimating governance performance where respondents wish to present their institutions favorably. Second, representation of woreda-level actors was limited (17.6% of quantitative respondents). Because qualitative data consistently showed that frontline implementation challenges role overlap, resource constraints, and reliance on informal coordination were most acute at lower administrative levels, the underrepresentation of woreda voices likely means quantitative domain scores underestimate the severity of implementation gaps. Third, the study sample was predominantly male (79.7%), which limits our ability to examine potential gender-based differences in governance perceptions or experiences. Future studies should intentionally oversample district-level actors and employ purposive gender-stratified sampling to obtain more representative and equitable estimates.

**Location:** Limitations (lines 514-526)

**Comment R2-5: Presentation and Flow – Tightening**

A small amount of tightening in a few descriptive sections could improve flow.

**Response:** We have carefully edited the manuscript for conciseness and readability without sacrificing scientific content.

**Revisions:**

- **Background section:** Reduced from approximately 1,200 to 950 words by consolidating repetitive descriptions of global frameworks.
- **Results section (qualitative findings):** Three longer paragraphs were split into shorter, more digestible paragraphs. Repetitive opening phrases were varied or removed.
- **Discussion section:** Two paragraphs that overlapped in content (coordination challenges and policy implementation) were merged.
- **Grammar and style:** Corrected minor issues including repetitive phrasing, run-on sentences, and inconsistent tense use.

**Location:** Throughout the manuscript

**Additional action taken**

We have also revised the title of Table 3 from “Mean Scores and Institutional Maturity Classification Across Governance Domains” to **“**Mean Perceived Scores and Descriptive Maturity Level Across Governance Domains**”** to align with the perceptual framing.

**Location:** Table 3

**Response to editorial request for marked-up and clean manuscripts**

We have uploaded the following files as requested:

1. Response to Reviewers (this document) – separate file
2. Revised Manuscript with Track Changes – showing all additions and deletions
3. Manuscript (clean version) – final, unmarked version

**CONCLUSION**

We are deeply grateful to the editor and both reviewers for their rigorous and constructive engagement with our work. The revisions have substantially improved the manuscript's methodological transparency, interpretive caution, and overall scientific quality. We believe the paper now meets the publication standards of *PLOS Global Public Health* and makes a meaningful contribution to the evidence base on multisector emergency governance in low-resource settings.

We look forward to your final decision.

Respectfully submitted,

**Sileshi Demelash Sasie, MPH, PhD**
Corresponding Author
Ethiopian Public Health Institute, Addis Ababa, Ethiopia
Email: sileyeshi21@gmail.com
ORCID: 0000-0002-7273-8923

**On behalf of all co-authors:**
Melkamu Asrat Alava, Lensa Fekadu, Haymanot Firdie, Neima Zeynu Ali, Tarekegn Solomon, Melkamu Abte Afele
