# Supplementary material for: Assessing the policy and institutional framework for multisectoral governance and accountability in emergency preparedness and response in Ethiopia
Source: PLOS Glob Public Health. 2026 Jul 24;6(7):e0006915. doi: 10.1371/journal.pgph.0006915 (PMC13399327; doi:10.1371/journal.pgph.0006915)
Supplement: Peer review history

# Peer review: Decision Letter 0

**DOI:** 10.1371/journal.pgph.0006915.r001

**Authors:** Julia Robinson

4 May 2026

PGPH-D-26-00736

Assessing the Policy and Institutional Framework for Multisectoral Governance and Accountability in Emergency Preparedness and Response in Ethiopia

PLOS Global Public Health

Dear Dr. Sasie,

Thank you for submitting your manuscript to PLOS Global Public Health. After careful consideration, we feel that it has merit but does not fully meet PLOS Global Public Health’s publication criteria as it currently stands. Therefore, we invite you to submit a revised version of the manuscript that addresses the points raised during the review process.

Please submit your revised manuscript by Jun 03 2026 11:59PM. If you will need more time than this to complete your revisions, please reply to this message or contact the journal office at globalpubhealth@plos.org. When you're ready to submit your revision, log on to https://www.editorialmanager.com/pgph/ and select the 'Submissions Needing Revision' folder to locate your manuscript file.

Please include the following items when submitting your revised manuscript:

- A letter that responds to each point raised by the editor and reviewer(s). You should upload this letter as a separate file labeled 'Response to Reviewers'.
- A marked-up copy of your manuscript that highlights changes made to the original version. You should upload this as a separate file labeled 'Revised Manuscript with Track Changes'.
- An unmarked version of your revised paper without tracked changes. You should upload this as a separate file labeled 'Manuscript'.

Guidelines for resubmitting your figure files are available below the reviewer comments at the end of this letter.

As the corresponding author, your ORCID iD is verified in the submission system and will appear in the published article. PLOS supports the use of ORCID, and we encourage all coauthors to register for an ORCID iD and use it as well. Please encourage your coauthors to verify their ORCID iD within the submission system before final acceptance, as unverified ORCID iDs will not appear in the published article. *Only*  the individual author can complete the verification step; PLOS staff *cannot*  verify ORCID iDs on behalf of authors.

We look forward to receiving your revised manuscript.

Kind regards,

Julia Robinson

Executive Editor

PLOS Global Public Health

**Journal Requirements:**

1. In the ethics statement in the Methods, you have specified that verbal consent was obtained. Please provide additional details regarding how this consent was documented and witnessed, and state whether this was approved by the IRB

2. We note that you have indicated that there are restrictions to data sharing for this study. For studies involving human research participant data or other sensitive data, we encourage authors to share de-identified or anonymized data. However, when data cannot be publicly shared for ethical reasons, we allow authors to make their data sets available upon request. For information on unacceptable data access restrictions, please see http://journals.plos.org/plosone/s/data-availability#loc-unacceptable-data-access-restrictions.

Before we proceed with your manuscript, please address the following prompts:

a) If there are ethical or legal restrictions on sharing a de-identified data set, please explain them in detail (e.g., data contain potentially identifying or sensitive patient information, data are owned by a third-party organization, etc.) and who has imposed them (e.g., a Research Ethics Committee or Institutional Review Board, etc.). Please also provide contact information for a data access committee, ethics committee, or other institutional body to which data requests may be sent.

b) If there are no restrictions, please upload the minimal anonymized data set necessary to replicate your study findings to a stable, public repository and provide us with the relevant URLs, DOIs, or accession numbers. Please see http://www.bmj.com/content/340/bmj.c181.long for guidelines on how to de-identify and prepare clinical data for publication. For a list of recommended repositories, please see https://journals.plos.org/plosone/s/recommended-repositories. You also have the option of uploading the data as Supporting Information files, but we would recommend depositing data directly to a data repository if possible.

Please update your Data Availability statement in the submission form accordingly.

3. Please ensure that your Ethics Statement is available in its entirety at the beginning of your Methods section, under a subheading 'Ethics Statement'.

4. We have noticed that you have uploaded Supporting Information files, but you have not included a list of legends. Please add a full list of legends for your Supporting Information files after the references list.

If the reviewer comments include a recommendation to cite specific previously published works, please review and evaluate these publications to determine whether they are relevant and should be cited. There is no requirement to cite these works unless the editor has indicated otherwise.

Please review your reference list to ensure that it is complete and correct. If you have cited papers that have been retracted, please include the rationale for doing so in the manuscript text, or remove these references and replace them with relevant current references. Any changes to the reference list should be mentioned in the rebuttal letter that accompanies your revised manuscript. If you need to cite a retracted article, indicate the article’s retracted status in the References list and also include a citation and full reference for the retraction notice.

**Additional Editor Comments (if provided):**

[Note: HTML markup is below. Please do not edit.]

Reviewers' comments:

Reviewer's Responses to Questions

**Comments to the Author**

1. Does this manuscript meet PLOS Global Public Health’s publication criteria? Is the manuscript technically sound, and do the data support the conclusions? The manuscript must describe methodologically and ethically rigorous research with conclusions that are appropriately drawn based on the data presented.

Reviewer #1: Yes

Reviewer #2: Yes

**********

2. Has the statistical analysis been performed appropriately and rigorously?

Reviewer #1: No

Reviewer #2: Yes

**********

3. Have the authors made all data underlying the findings in their manuscript fully available (please refer to the Data Availability Statement at the start of the manuscript PDF file)?

The PLOS Data policy requires authors to make all data underlying the findings described in their manuscript fully available without restriction, with rare exception. The data should be provided as part of the manuscript or its supporting information, or deposited to a public repository. For example, in addition to summary statistics, the data points behind means, medians and variance measures should be available. If there are restrictions on publicly sharing data—e.g. participant privacy or use of data from a third party—those must be specified.

Reviewer #1: Yes

Reviewer #2: Yes

**********

4. Is the manuscript presented in an intelligible fashion and written in standard English?

PLOS Global Public Health does not copyedit accepted manuscripts, so the language in submitted articles must be clear, correct, and unambiguous. Any typographical or grammatical errors should be corrected at revision, so please note any specific errors here.

Reviewer #1: Yes

Reviewer #2: Yes

**********

5. Review Comments to the Author

Please use the space provided to explain your answers to the questions above. You may also include additional comments for the author, including concerns about dual publication, research ethics, or publication ethics. (Please upload your review as an attachment if it exceeds 20,000 characters)

**Reviewer #1:**  This is a well-conceived and methodologically sound mixed-methods study addressing an important but under-evidenced area: how multisector emergency governance functions in actuality in low-income settings. The manuscript is well-structured, and could make a valuable contribution in health system of Ethiopia. Some aspects of the manuscript need attention before publication.

1. There seems to be a conceptual and analytical overclaim regarding the ‘maturity’ framework. Authors calculate a mean overall governance maturity score of 3.31 (‘functional’) but provide no validation or justification for the specific 1-5 thresholds used. The categories (nascent, emerging, functional, optimized) are described as ‘a priori’ but referenced to an unpublished national framework, which is insufficient. Without evidence that a 3.0 cutoff meaningfully distinguishes ‘functional’ from ‘emerging’ in this context, the scoring risks appearing arbitrary. Therefore, I would suggest grounding the cutoffs in published precedent, OR re-frame the scores as descriptive averages without maturity labeling, OR move the maturity interpretation to the discussion as heuristic rather than a finding of your study.

2. Methods: Target sample of 80 was calculated using a 10% margin of error and 90% confidence, unusually low precision for a quantitative governance assessment. Moreover, only 13 of 74 respondents (17.6%) were from ‘woreda’ (district) level, yet qualitative findings repeatedly claim subnational governance is weakest. This risks federal/regional bias. You do acknowledge this briefly but should state more directly that ‘quantitative domain scores are not reliably representative of frontline perspectives.’

3. Results:

a) You presented quantitative scores (Table 3) and qualitative themes separately, then the discussion integrates them thematically. However, there is no systematic ‘joint display’ or cross-tabulation showing, for example, how respondents who rated ‘policy frameworks’ as emerging differed in their qualitative accounts from those rating it higher. Without this, the claim of ‘sequential mixed-methods integration’ is not fully realized.

b) All quantitative measures are Likert-scale perceptions of governance, not independent assessments (e.g., after-action review scores, documented reporting timeliness, or simulated exercise performance). You do note this in limitations but then draw relatively strong conclusions about ‘functional’ governance. I recommend that you rephrase it both in the abstract and conclusion to specify that findings reflect ‘perceived’ governance maturity, not objectively measured performance.

c) Table 1: Gender distribution (79.7% male) is striking but not discussed. A sentence on potential gender bias in governance perceptions or inclusion would strengthen reflexivity.

d) Comparison to Ebola and COVID-19 coordination challenges is helpful but currently very brief. You could expand by one sentence specifying which coordination failures (e.g., role ambiguity, parallel reporting) mirror the Ethiopian findings.

4. References: Several citations are incomplete or need formatting according to the journal’s style and requirement. There is a recent paper published in the same journal which could be an interesting reference to cite. https://pubmed.ncbi.nlm.nih.gov/41920850/

With these adjustments, the paper will be a strong contribution to global public health governance literature.

**Reviewer #2:**  Date 5/4/2026

Manuscript number =PGPH-D-26-00736

Article type =Research Article

Short Title =Multisectoral Governance and Accountability in Emergency Preparedness in Ethiopia

Reviewer= Abiti A.

Email: asamnewabity@gmil.com

Comments and recommendation

Summary

This is a strong and carefully conducted study that addresses an important gap in understanding how multisectoral governance for emergency preparedness actually works in practice. The combination of quantitative assessment with qualitative insights adds real depth to the analysis, and the manuscript is clearly grounded in both national context and global frameworks. The work is timely, relevant, and offers practical value for policymakers and implementers. Overall, the paper is well written and makes a meaningful contribution. However, if the points outlined below are taken into consideration, the manuscript would be further strengthened and its clarity and impact enhanced.

Comment

1. Background

The background is comprehensive and well contextualized. It would be helpful, however, to more clearly state the specific gap this study is addressing, particularly in terms of what is not yet understood about how governance structures function operationally, beyond what is already described in policies and frameworks.

Comments

2. Methods

The methods are appropriate and generally clear. A short clarification on how the seven governance domains were defined and how the indicators were assigned to each domain would improve transparency and make the approach easier to apply in other settings.

3. Results

The results are rich and detailed. Adding a brief summary paragraph at the beginning of the Results section to highlight the main patterns would help guide the reader before moving into the domain-by-domain findings.

4. Limitations

The limitations are acknowledged, which is appreciated. It would strengthen the discussion to briefly reflect on how reliance on self-reported data and limited representation at woreda level might influence the findings, especially in relation to actual implementation at frontline level.

5. Presentation and flow

The manuscript reads well overall. A small amount of tightening in a few descriptive sections could improve flow, but this is minor and does not affect the quality of the work.

Overall Assessment

This is a well-prepared and thoughtful manuscript. With minor refinement, it is suitable for publication and will be useful for both national and global audiences working on public health emergency preparedness.

To the Editor

This is a solid and credible study with clear relevance to current global health priorities. The analysis is sound and the findings are meaningful. I recommend acceptance after minor revision

Recommendation

Minor revision

**********

6. PLOS authors have the option to publish the peer review history of their article (what does this mean?). If published, this will include your full peer review and any attached files.

**Do you want your identity to be public for this peer review?**  If you choose “no”, your identity will remain anonymous but your review may still be made public.

For information about this choice, including consent withdrawal, please see our Privacy Policy.

Reviewer #1: **Yes:**  Babar Tasneem Shaikh

Reviewer #2: No

**********

[NOTE: If reviewer comments were submitted as an attachment file, they will be attached to this email and accessible via the submission site. Please log into your account, locate the manuscript record, and check for the action link "View Attachments". If this link does not appear, there are no attachment files.]

**Figure Resubmissions:**

While revising your submission, we strongly recommend that you use PLOS’s NAAS tool (https://ngplosjournals.pagemajik.ai/artanalysis) to test your figure files. NAAS can convert your figure files to the TIFF file type and meet basic requirements (such as print size, resolution), or provide you with a report on issues that do not meet our requirements and that NAAS cannot fix.

After uploading your figures to PLOS’s NAAS tool - https://ngplosjournals.pagemajik.ai/artanalysis, NAAS will process the files provided and display the results in the "Uploaded Files" section of the page as the processing is complete. If the uploaded figures meet our requirements (or NAAS is able to fix the files to meet our requirements), the figure will be marked as "fixed" above. If NAAS is unable to fix the files, a red "failed" label will appear above. When NAAS has confirmed that the figure files meet our requirements, please download the file via the download option, and include these NAAS processed figure files when submitting your revised manuscript.

# Author comment: Author response to Decision Letter 1

**DOI:** 10.1371/journal.pgph.0006915.r002

23 May 2026

Attachment Submitted filename: Point by point response.docx

# Peer review: Decision Letter 1

**DOI:** 10.1371/journal.pgph.0006915.r003

**Authors:** Veena Sriram

7 Jul 2026

Assessing the Policy and Institutional Framework for Multisectoral Governance and Accountability in Emergency Preparedness and Response in Ethiopia

PGPH-D-26-00736R1

Dear Dr Sasie,

We are pleased to inform you that your manuscript 'Assessing the Policy and Institutional Framework for Multisectoral Governance and Accountability in Emergency Preparedness and Response in Ethiopia' has been provisionally accepted for publication in PLOS Global Public Health.

Before your manuscript can be formally accepted you will need to complete some formatting changes, which you will receive in a follow up email. A member of our team will be in touch with a set of requests.

Please note that your manuscript will not be scheduled for publication until you have made the required changes, so a swift response is appreciated.

IMPORTANT: The editorial review process is now complete. PLOS will only permit corrections to spelling, formatting or significant scientific errors from this point onwards. Requests for major changes, or any which affect the scientific understanding of your work, will cause delays to the publication date of your manuscript.

If your institution or institutions have a press office, please notify them about your upcoming paper to help maximize its impact. If they'll be preparing press materials, please inform our press team as soon as possible -- no later than 48 hours after receiving the formal acceptance. Your manuscript will remain under strict press embargo until 2 pm Eastern Time on the date of publication. For more information, please contact globalpubhealth@plos.org.

Thank you again for supporting Open Access publishing; we are looking forward to publishing your work in PLOS Global Public Health.

Best regards,

Veena Sriram

Academic Editor

PLOS Global Public Health

***********************************************************

Some minor typos present that can be addressed during the proofing stage.

Reviewer Comments (if any, and for reference):

Reviewer's Responses to Questions

**Comments to the Author**

1. If the authors have adequately addressed your comments raised in a previous round of review and you feel that this manuscript is now acceptable for publication, you may indicate that here to bypass the “Comments to the Author” section, enter your conflict of interest statement in the “Confidential to Editor” section, and submit your "Accept" recommendation.

Reviewer #1: All comments have been addressed

Reviewer #2: (No Response)

**********

2. Does this manuscript meet PLOS Global Public Health’s publication criteria? Is the manuscript technically sound, and do the data support the conclusions? The manuscript must describe methodologically and ethically rigorous research with conclusions that are appropriately drawn based on the data presented.

Reviewer #1: Yes

Reviewer #2: Yes

**********

3. Has the statistical analysis been performed appropriately and rigorously?

Reviewer #1: Yes

Reviewer #2: Yes

**********

4. Have the authors made all data underlying the findings in their manuscript fully available (please refer to the Data Availability Statement at the start of the manuscript PDF file)?

The PLOS Data policy requires authors to make all data underlying the findings described in their manuscript fully available without restriction, with rare exception. The data should be provided as part of the manuscript or its supporting information, or deposited to a public repository. For example, in addition to summary statistics, the data points behind means, medians and variance measures should be available. If there are restrictions on publicly sharing data—e.g. participant privacy or use of data from a third party—those must be specified.

Reviewer #1: Yes

Reviewer #2: Yes

**********

5. Is the manuscript presented in an intelligible fashion and written in standard English?

PLOS Global Public Health does not copyedit accepted manuscripts, so the language in submitted articles must be clear, correct, and unambiguous. Any typographical or grammatical errors should be corrected at revision, so please note any specific errors here.

Reviewer #1: Yes

Reviewer #2: Yes

**********

6. Review Comments to the Author

Please use the space provided to explain your answers to the questions above. You may also include additional comments for the author, including concerns about dual publication, research ethics, or publication ethics. (Please upload your review as an attachment if it exceeds 20,000 characters)

Reviewer #1: The authors have been responsive, thorough, and scientifically rigorous in their revisions. The manuscript now meets the methodological and transparency standards expected by PLOS Global Public Health.

Reviewer #2: Thanks for addressing all comments

**********

7. PLOS authors have the option to publish the peer review history of their article (what does this mean?). If published, this will include your full peer review and any attached files.

**Do you want your identity to be public for this peer review?**  If you choose “no”, your identity will remain anonymous but your review may still be made public.

For information about this choice, including consent withdrawal, please see our Privacy Policy.

Reviewer #1: **Yes:**  Babar Tasneem SHAIKH

Reviewer #2: No

**********
